# P007 Delayed-Onset Acute Anterior Uveitis Associated with Long-Term Zoledronic Acid Therapy in Metastatic Prostate Cancer

**DOI:** 10.1093/rap/rkag113.037

**Published:** 2026-11-03

**Authors:** Khin Swe Win, Khaled Mahmoud, Khizer Iqbal, Zunaid Karim

**Affiliations:** Mid Yorkshire Teaching Hospital Trust, Wakefield, United Kingdom; Mid Yorkshire Teaching Hospital Trust, Wakefield, United Kingdom; Mid Yorkshire Teaching Hospital Trust, Wakefield, United Kingdom; Mid Yorkshire Teaching Hospital Trust, Wakefield, United Kingdom

## Abstract

**Introduction:**

Bisphosphonates are indicated for osteoporosis, hypercalcaemia of malignancy, and Paget’s disease of bone by improving bone strength and relieving bone pain. Zoledronic acid, an intravenous bisphosphonate, reduces skeletal-related events in metastatic prostate cancer. Common adverse effects include flu-like symptoms, gastrointestinal symptoms, musculoskeletal pain, and acute kidney injury [1]. However, ocular complications are uncommon and often overlooked. Acute anterior uveitis (AAU) is a rare, sight-threatening adverse effect that requires prompt recognition and treatment. We present a case of delayed-onset unilateral AAU in a patient receiving long-term zoledronic acid for metastatic prostate cancer, highlighting the importance of awareness of this rare complication.

**Case description:**

A 77-year-old male with metastatic prostate adenocarcinoma presented with acute left eye pain and reduced vision. There was no history of prior eye disease. He was diagnosed with prostate cancer in 2023, and in April 2025, he was found to have bony metastases involving the right anterior pubic bone, inferior sacrum, and right proximal femur.

The patient was commenced on systemic chemotherapy and intravenous zoledronic acid (Zometa) in June 2025 for the management of metastatic bone disease. Chemotherapy was discontinued in November 2025, prior to cycle seven, following a non-pathological fracture of the left clavicle; however, zoledronic acid infusions were continued every 3–4 weeks.

In April 2026, approximately 11 months after the initiation of zoledronic acid therapy, he presented with a painful left eye associated with reduced visual acuity. Ophthalmic examination demonstrated acute anterior uveitis of the left eye with a small degree of vitreous spillover.

A diagnosis of left acute anterior uveitis was made following ophthalmology assessment. The patient was treated with topical corticosteroid therapy, resulting in improvement of symptoms and ocular inflammation. One week later, he also developed herpes zoster involving the left periocular region, which complicated the clinical course and required further assessment and management.

Given the temporal relationship between prolonged zoledronic acid treatment and the onset of ocular inflammation, zoledronic acid-induced acute anterior uveitis was considered the most likely diagnosis. The patient remains under ophthalmology follow-up for monitoring of inflammatory resolution and visual recovery.

**Discussion:**

Acute anterior uveitis is a recognised but uncommon adverse effect of bisphosphonate therapy. It can be unilateral or bilateral. [2] Prompt recognition is essential, as delayed diagnosis may lead to significant patient discomfort and potential visual morbidity.

The exact pathogenesis of zoledronic acid-induced acute anterior uveitis remains incompletely understood. It is thought that nitrogen-containing bisphosphonates activate gamma delta T lymphocytes through inhibition of the mevalonate pathway, leading to the release of pro-inflammatory cytokines, including tumour necrosis factor-α (TNF-α), interleukin-1 (IL-1), and interleukin-6 (IL-6). This acute inflammatory response is believed to account for the typical onset of ocular inflammation within 24–72 hours following the initial infusion. However, the mechanism underlying delayed-onset reactions remains uncertain. It has been postulated that factors such as the physicochemical properties of the drug and prolonged tissue deposition may contribute to delayed ocular inflammation, although definitive evidence is lacking. [3]

In this case, the patient developed unilateral acute anterior uveitis after approximately 11 months of treatment with zoledronic acid. Most reported cases in the literature occur within a few days of the first infusion, making this delayed presentation unusual and potentially more difficult to attribute to the medication. [4] The absence of other clear precipitating factors and the temporal association with ongoing zoledronic acid therapy supported the diagnosis of drug-induced ocular inflammation.

The patient responded well to topical corticosteroid treatment, which remains the mainstay of management for bisphosphonate-associated uveitis.

This case highlights the need for greater awareness of ocular adverse effects among rheumatologists, oncologists, ophthalmologists, and primary care clinicians. Patients receiving zoledronic acid should be advised to seek urgent medical attention if they develop eye pain, redness, photophobia, or visual disturbance. Earlier recognition may facilitate prompt treatment and improve visual outcomes.

**Key learning points:**

• Acute anterior uveitis is a rare but important ocular adverse effect of zoledronic acid therapy.

• Although most reported cases occur within 24–72 hours of the first infusion, clinicians should be aware that delayed presentations can occur even after prolonged treatment.

• Patients receiving zoledronic acid who develop eye pain, redness, photophobia, or reduced vision require urgent ophthalmological assessment.

• Early diagnosis and treatment with topical corticosteroids can result in rapid symptom improvement and reduce the risk of visual complications.

• Zoledronic acid remains an important treatment for metastatic bone disease, reducing skeletal-related events, improving bone strength, and providing symptomatic relief from bone pain. However, clinicians should remain vigilant for uncommon ocular adverse effects.

• By reviewing patient information leaflets from several NHS trusts, only Leeds and Norfolk were found to list uveitis as a side effect of the medication. Alder Hey Children’s NHS Foundation Trust mentioned eye inflammation as a warning.

